# COVID-19 and Fatty Liver Disorders

**DOI:** 10.3390/jcm12134316

**Published:** 2023-06-27

**Authors:** Maria Guarino, Valentina Cossiga, Francesco Maria Cutolo, Maria Rosaria Attanasio, Raffaele Lieto, Filomena Morisco

**Affiliations:** Diseases of the Liver and Biliary System Unit, Department of Clinical Medicine and Surgery, University of Naples “Federico II”, 80131 Naples, Italy

**Keywords:** NAFLD, MAFLD, COVID-19, obesity, fibrosis, telemedicine, vaccination

## Abstract

In late 2019, the world was shaken by the COVID-19 pandemic. Severe Acute Respiratory Syndrome Coronavirus—2 (SARS-CoV-2) infection became one of the main causes of illness and hospitalization worldwide, especially in subjects with metabolic comorbidities such as obesity, diabetes, or liver disease. This scenario crosses with the metabolic liver disorders’ “pandemic”, caused by the exponential spreading of non-alcoholic fatty liver disease, which is now the most prevalent cause of chronic liver disease (CLD). The aim of this review is to analyze the key factors of the relationship between COVID-19 and the spectrum of fatty liver disorders (FLD), in terms of molecular mechanisms and clinical presentation which can predict a more severe course of the infection. In addition, this review will face the change in management of FLD during pandemics, with a central role of telemedicine, and the role of other interventions in preventing and treating severe infection in these subjects.

## 1. Introduction

COVID-19 is an infectious disease caused by Severe Acute Respiratory Syndrome Coronavirus—2 (SARS-CoV-2). In these last 3 years, the COVID-19 pandemic has caused many issues worldwide, primarily in the healthcare field but also in the economical, sociological, and political spheres. It is well known that patients with chronic liver disease (CLD) have an increased risk of adverse outcomes [1,2,3].

Non-alcoholic fatty liver disease (NAFLD) is nowadays the most CLD in the Western population and non-alcoholic steatohepatitis (NASH) is its progressive form from fibrosis to cirrhosis and its complications [4]. Recently a new definition has been proposed: metabolic dysfunction-associated fatty liver disease (MAFLD), that can be diagnosed with histological, imaging, or blood biomarkers of liver fat accumulation in addition to one of the following three criteria: overweight/obesity, presence of type 2 diabetes mellitus, or evidence of metabolic dysregulation [5].

This review aims to give an overview of the relationship between COVID-19 and the spectrum of all fatty liver disorders (FLD) (*including in this definition NAFLD, MAFLD, and NASH*), evaluating how the pandemic has changed FLD epidemiology and how FLD has determined a more severe course of infection. Other investigated topics are the change in management of FLD during pandemic, with the role of telemedicine, and the main strategies in preventing and treating patients with a higher risk of severe infection.

## 2. Epidemiology

### 2.1. FLD Epidemiology during COVID-19 Pandemic

Prevalence of FLD has increased in Western countries in the last 10 years, sustained by the elevated rates of obesity and diabetes, as shown by several studies highlighting a relevant change in NAFLD prevalence from the 1990s to 2010s [6,7,8]. Then, before the SARS-CoV-2 pandemic, NAFLD prevalence was about 21–27% in North America [9], 30.45% in South America, 23.71% in Europe [10], 42% in China [11], and 8.7% in sub Saharan Africa [12]. By the end of March 2020, because of the introduction in several countries of total or partial lockdown to halt the spread of COVID-19, the overall activity time has fallen and also eating habits have changed, influencing the prevalence of FLD.

A longitudinal study on the impact of the COVID-19 lockdown on NAFLD in a Spanish population of 6236 workers underlines a 12.59% increased risk of developing NAFLD according to the fatty liver index (FLI) compared to the pre-lockdown value [13].

Another Japanese retrospective longitudinal study on 973 subjects undergoing health check-ups between 2018 and 2020 shows an increased rate of MAFLD diagnosis. In fact, in 2018, 27% of participants were diagnosed with MAFLD; in 2019, 22 new patients of this cohort developed MAFLD; and in 2020, another 44 subjects received the same diagnosis. This study also reported that all subjects routinely eating late-night meals had a higher risk of MAFLD, highlighting the importance of lifestyle changes during pandemics in MAFLD prevalence [14].

There are also worrying data about FLD in youth. A retrospective cross-sectional study from the Yale Study of the Pathophysiology of Prediabetes/Type 2 Diabetes in youth showed an increased prevalence of NAFLD in adolescents with obesity (from 37.3% in 2017–2019 to 60.9% in 2020–2022) with a worsening of their metabolic status [15]. Accordingly, several studies showed an increase in body weight, glycemia, transaminases, and intrahepatic fat content in subjects during pandemic, with a growing risk of progression to NASH and fibrosis [16,17].

Despite the efforts of including people with FLD in a pre-lockdown interventional program, the pandemic also determined a worsening in similar strategies. In an Italian cohort of 41 people with NAFLD enrolled in an educational program of 12 months, after an initial body weight loss, a significant weight gain was seen in 70% of them during the pandemic months [18].

Furthermore, the rates of first liver-related events (LRE) in people with cirrhosis due to NAFLD has grown from 7.4% to 11.3% in the pandemic periods, but the worsened metabolic status did not seem to be associated with a higher risk of LRE, since at multivariable analyses, only diabetes, albumin levels, and Fibrosis-4 index (FIB-4) were associated with a higher risk [19].

Finally, the COVID-19 pandemic also had an important impact on hepatocellular carcinoma (HCC) incidence and severity [1]. A Northern English retrospective observational study highlights a rise in the incidence of HCC and a worse tumor burden with lower HCC diagnosis under surveillance [20]. This study underlines that NAFLD was the most common underlying cause of HCC in 2020–2021. This could be ascribed to the delay in the surveillance and treatment programs due to lockdown.

### 2.2. The Incidence of COVID-19 in Subjects with FLD

Some studies highlight a higher cumulative incidence of COVID-19 in patients with FLD. Ghoneim et al., extrapolated the health records of 61.4 million adults from a big commercial database including 26 healthcare systems in the USA, to identify subjects with a diagnosis of SARS-CoV-2 infection from December 2019 to May 2020. The odds of having COVID-19 were higher in patients with NASH [21].

Another study, on a cohort of 230 subjects, enrolled from the Health Treatment Center of Changsha after a laboratory confirmed diagnosis of COVID-19, highlighted a higher incidence in patients with FLD than in the general population [22]. Also in South Korea, Hae Won Yoo et al., evaluated people with positive laboratory tests for SARS-CoV-2 from national COVID-19-related registers to analyze the association with NAFLD. Patients were divided into three groups, according to three different FLD definition: (1) people with high levels of the hepatic steatosis index (HSI) (≥36); (2) FLI (≥60); or (3) a claim-based definition indicated by the International Classification of Diseases, 10th revision (ICD-10). Each group had a higher risk of COVID-19 infection and of more severe illness, and this risk is even higher in cases of advanced fibrosis estimated using the BARD score (a simple score based on the body mass index (BMI), aspartate aminotransferase (AST)/alanine aminotransferase (ALT) ratio, and presence of type 2 diabetes) [23].

## 3. Pathogenesis of Systemic and Liver Damage

Since the beginning of the pandemic in 2020, several studies have demonstrated a relationship between FLD and the susceptibility to SARS-CoV-2 infection with a higher severity of illness. However, the molecular mechanisms at the basis of these relationships have been partially elucidated.

Regarding the virus entry in human cells, the key proteins seem to be angiotensin-converting enzyme 2 (ACE-2), the functional receptor for virus spike (S) protein expressed in many organs (including lung and liver), and the transmembrane serine-protease 2 (TMPRSS2), which is responsible for the S protein priming after entry [24]. According to an Italian study, ACE-2 and TMPRSS2 liver tissue expression levels are increased in NAFLD patients compared to those with viral hepatitis [25]. Additionally, it seems that the basal expression of ACE-2 is significantly higher in NAFLD, so the increased number of available cellular receptors facilitates viral entry [26]. Still, according to genetic studies, human genes implicated in the replication and pathogenesis of SARS-CoV-2 are positively regulated in NAFLD, with increased activation of the JAK (Janus kinase)/STAT (signal transducers and activators of transcription) signaling pathway inducing overexpression of the interferons encoding genes, or the IL-6 overexpression occurring via tumor necrosis factor (TNF) and nuclear factor kappa B (NF-κB) signaling pathways. Additionally, the increased expression of Furin and TMPRSS2 facilitates the cytokine storm in increasing the infection severity [27]. All these factors can explain the greater susceptibility of patients with FLD to SARS-CoV-2 infection.

Several studies have shown that patients with FLD and SARS-CoV-2 infection may have a mild to moderate liver injury with a predominant cytotoxic pattern (AST and ALT elevation) and rarely with the cholestatic one, in this case with a more severe COVID-19 infection [27,28,29,30,31,32]. The transient and mild hepatocellular injury can be explained by the study of Chai et al., demonstrating that ACE-2 receptors in the liver are expressed mostly in cholangiocytes and less in hepatocytes, suggesting that hepatocytes might not be targeted by the virus, or at least not through ACE-2, and probably the cytotoxic liver damage is caused by drugs or by the systemic inflammatory response induced by COVID-19 [33]. Elevation of gamma-glutamyl transferase (GGT) in patients with FLD is also described [30,32] but it can just be a part of the primary liver disease. Moreover, a retrospective study by Tripon et al., suggested the use of a liver test at admission to evaluate the risk of COVID-19 disease progression, since a hepatocellular pattern 2 × upper limit of normal (ULN) correlated with the severity of the infection [34].

## 4. Severity of COVID-19 in Subjects with FLD

Regarding the increased severity of SARS-CoV-2 infection in subjects with FLD, some progress has been made. Already in the pre-pandemic era, a more severe community-acquired pneumonia in NAFLD patients was highlighted, in terms of a higher need for invasive mechanical ventilation, respiratory extracorporeal membrane oxygenation, and continuous renal replacement therapy [35]. Regarding SARS-CoV-2 infection, with or without pneumonia, current data seem to confirm this increased severity.

### 4.1. COVID-19 and Steatosis

The association between COVID-19 and the spectrum of steatotic liver disease has been extensively studied in recent years, with conflicting results.

Recently, in a prospective Croatian study, NAFLD patients with severe SARS-CoV-2 infection (bilateral pulmonary infiltrates on chest imaging, SpO2 ≤ 94%, and/or dyspnea) presented a more distinct serum cytokine profile than patients without NAFLD. In detail, increasing levels of pro-inflammatory cytokines have been observed, such as interleukin 6 (IL-6), described as an independent prognostic factor for COVID-19 mortality [36], interleukin 8 (IL-8), interferon-gamma (IFN-γ)-induced protein 10 kDa (IP-10), or C–X–C motif chemokine 10 (CXCL10); and lower levels of IFN- γ [37]. Of interest, the decreased IFN-γ concentrations are most likely associated with the functional impairment of Natural Killer cells, thus explaining, together with the altered concentrations of B cells and T cells and with the altered levels of the immunomodulatory cytokine interleukin 10 (IL-10), the dysregulation of cell-mediated immunity [38]. Still, according to a Chinese study, MAFLD subjects have significantly higher circulating IL-6 levels than non-MAFLD, and there is a significant interaction between elevated IL-6 levels with severe COVID-19 in MAFLD. Based on these data, it is possible to hypothesize that the presence of MAFLD can exacerbate the virus-induced cytokine ‘storm’, possibly through the hepatic release of multiple pro-inflammatory cytokines, including IL-6 (whose levels are basically elevated in MAFLD) [39].

A greater severity of infection in patients with FLD can explain the slightly increased age-standardized mortality, according to the database from the Centers for Disease Control and Prevention (CDC), although it is necessary to take into account the metabolic comorbidities (obesity, type 2 diabetes, hypertension) of these patients [40]. Several retrospective studies demonstrated a high rate of severe COVID-19 in hospitalized patients with FLD, in terms of major need of O_2_ therapy, non-invasive ventilation, longer hospital stay [32], and also a more severe course with increased intensive-care-unit (ICU) admission, higher risk of intubation and mortality [41,42]. Other retrospective studies demonstrated that, in patients with normal BMI [22], and regardless of comorbidities [43], those with NAFLD who were infected by COVID-19 may have a higher proportion of developing severe disease; probably, the immune dysregulation descripted above plays a role even in lean NAFLD subjects. The increased risk seems to be gender related (major in men) [43,44].

On the other hand, contrary to what has been said so far, some studies do not define NAFLD as an independent risk factor for severe COVID-19 [45,46,47]. Forlano et al., in a retrospective UK study, demonstrated that NAFLD is associated with adverse outcomes in patients hospitalized for COVID-19 only in association with very elevated inflammatory markers (especially C-reactive protein, ferritin, and the clinical score EWS—early warning score) [45]. Still, according to an Indian study, in a large cohort of patients with SARS-CoV-2 infection, NAFLD was not a predictor of severe disease [46], and even in a Mexican study, in patients with steatosis assessed by HSI, a worsening of the outcomes from COVID-19 has not been demonstrated [47].

Among the doubts on the real role of fatty liver disease *per se*, many studies emphasized the role of metabolic comorbidities. About metabolic comorbidities, some of these, i.e., diabetes and visceral obesity have to be highlighted for their prevalence in the general population. According to the Mexican study mentioned above, obese patients face an increased risk of severe complications and mortality [47]; furthermore, Tignanelli et al., found that treatments for metabolic syndrome greatly mitigated the risk from COVID-19 [44]. In their analysis, NAFLD/NASH subjects underwent bariatric surgery, or on treatment with metformin or glucagon-like peptide 1 receptor agonists (GLP-1RA) or statins, showed a significant decrease in the odds of hospitalization [44]. Furthermore, Jiuling Li et al., demonstrated the linkage between the increased severity of SARS-CoV-2 infection and BMI, waist circumference, and hip circumference, regardless of the presence of NAFLD [48]. This findings may be explained by the low-grade inflammation of obese subjects, due to the expansion of white visceral adipose tissue and to the release of cytokines and chemotactic factors such as platelet-derived growth factor (PDGF), monocyte chemoattractant protein-1 (MCP-1), transforming growth factor-β (TGF-β), interleukin-1β (IL-1β), IL-6, and tumor necrosis factor-α (TNFα) [49].

An interesting prognostic model in MAFLD patients with SARS-CoV-2 infection has been developed by Macías-Rodríguez et al.; this model, called the liver fibrosis and nutrition (LFN)-COVID-19 index, is based on lactate dehydrogenase and transaminases (AST/ALT), and shows a good performance to predict poor outcomes (acute kidney injury, intubation, death) when the value is >1.67. Nevertheless, further validation for this score is needed to include it in routine clinical practice [50].

### 4.2. COVID-19 and Liver Fibrosis

Another important factor to take into account in patients with FLD is the degree of hepatic fibrosis. A large part of the literature in this context evaluated fibrosis through non-invasive methods and, among these, the most studied is certainly the FIB-4. The FIB-4 model was originally developed to predict liver fibrosis in patients with HIV/HCV coinfection, using routine tests (AST, ALT, platelets) [51]; nowadays, it is extensively used for the non-invasive estimation of the liver fibrosis in hepatitis B, hepatitis C, and FLD subjects.

Several authors have demonstrated a role of FIB-4 in predicting the need for ICU admission, the rate of intubations, and the mortality for COVID-19 [28,41,52,53,54]; according to these studies, FIB-4 has a predictive value (although different cut-offs were used) just like other factors, i.e., age [55], diabetes [54], or leucocyte count [53]. Another model for the non-invasive assessment of fibrosis is the NAFLD-fibrosis score (NFS); according to a Chinese study, NAFLD patients with an NFS score ≥ –1.5 are at higher risk of severe COVID-19 [56].

All these data are in line with the higher rates of short-term mortality in cirrhotic patients previously reported by an Italian study [57]; however, it has to be noticed that in all these studies, the fibrosis was not evaluated with invasive methods or with liver stiffness measurement, so the real association between a high degree of liver fibrosis and COVID-19 severity deserves further studies.

**Figure 1:**
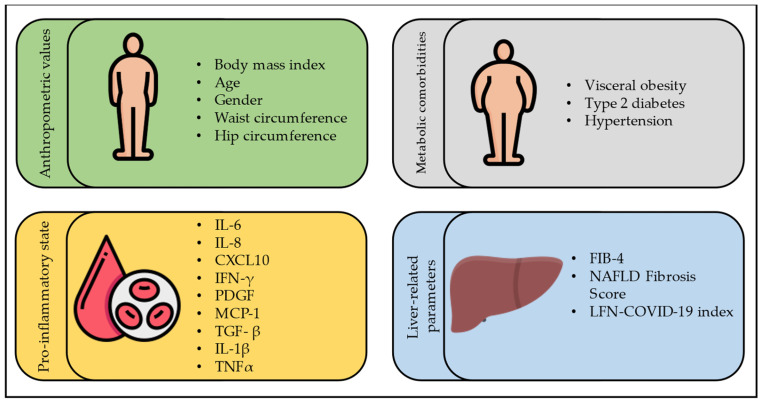
**Risk factors for COVID-19 severity in subjects with FLD.** CXCL10: C-X-C motif chemokine ligand 10; IFN-γ: interferon-gamma; IL-1β: interleukin-1beta; IL-6: interleukin-6; IL-8: interleukin-8; LFN-COVID-19 index: liver fibrosis and nutrition COVID-19 index; MCP-1: monocyte chemoattractant protein-1; PDGF: platelet-derived growth factor; TGF- β: transforming growth factor-beta; TNFα: tumor necrosis factor-alpha, FIB-4: Fibrosis-4 index.

In conclusion, we can confirm a strong association between FLD and severity of SARS-CoV-2 infection, in terms of lung and liver involvement, maybe for the presence of an inflammatory liver disease, which increases the risk of the ‘cytokine storm’. Of interest, the main factors associated with worse outcomes seem to be some elements of metabolic syndrome (obesity and diabetes above all) and the high degree of liver fibrosis (see Figure 1).

## 5. Management of COVID-19 in Subjects with FLD

**Figure 2:**
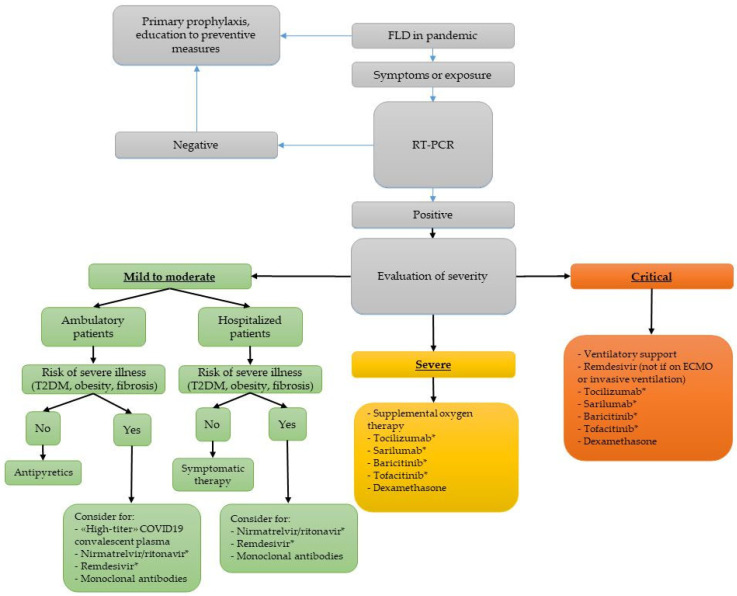
**Management of COVID-19 in subjects with FLD.** RT-PCR: real-time polymerase chain reaction, T2DM: type 2 diabetes mellitus, ECMO, extracorporeal membrane oxygenation. * Monitor liver blood tests.

According to World Health Organization (WHO) recommendations [58], there are different medical approaches for the management of COVID-19 on the basis of symptoms, comorbidities, and laboratory tests. In Figure 2, we report a possible flowthere are inconsistencies in use of capitals, e.g., body mass index, in figure text. Please adjust if can.chart approach to FLD. Patients with FLD are more likely to have abnormal transaminases and liver damage due to SARS-CoV-2 infection [27,28,29,30,31,32] and the presence of fibrosis is a negative prognostic factor [28,41,52,53,54,56]. However, on 5 May 2023, the WHO declared the end of COVID-19 as a global health emergency and all requirements to wear a mask have been lifted, except for some hospital wards with extremely frail patients, in nursing homes and in long-term care and rehabilitation centers. Nevertheless, patients with advanced fibrosis or cirrhosis, because of their higher risk of worse outcomes, should be encouraged to continue practicing preventive measures such as wearing a high-quality mask, especially in crowded indoor spaces and in clinical areas, in order to decrease the risk of infection.

### 5.1. Clinical Intervention and Treatment

For COVID-19-positive subjects, the severity classification recognizes the following: (1) mild disease if there are symptoms without pneumonia or hypoxia, (2) moderate disease if there are clinical signs of pneumonia, (3) severe disease if there is respiratory distress or SpO2 < 90% on room air, (4) critical disease in the presence of acute respiratory distress syndrome, sepsis, or septic shock [58]. 

Among patients with mild disease, the WHO guidelines suggest antipyretics for fever and pain, adequate nutrition, and appropriate rehydration, but subjects with risk factors for severe illness should be monitored closely, given the possible risk of deterioration. Then, subjects with FLD, especially with comorbidities such as diabetes or severe obesity or fibrosis, should be considered for high-titer COVID-19 convalescent plasma, nirmatrelvir/ritonavir, three-day treatment with remdesivir, or neutralizing monoclonal antibodies in agreement with the Infectious Disease Society of America (IDSA) 2022 guidelines [59].

In patients treated with remdesivir, the potential risk of hepatotoxicity could be a problem in patients with FLD, especially in a setting of liver inflammation and fibrosis. Several studies have highlighted that there are no absolute contraindications if liver function is closely monitored during treatment [60,61]. Despite a few case reports of suspected remdesivir-associated acute liver failure [62,63,64], IDSA guidelines suggest the administration of remdesivir also in hospitalized patients with mild to moderate COVID-19 with a high risk of progression and in patients with severe COVID-19, paying attention to the liver function test. In ambulatory patients, ritonavir could also induce mild to moderate elevation in aminotransferases and cholestasis [65,66]. 

Then, patients with FLD and COVID-19 mild infection can benefit by telemedicine tools, including sensors for continuous vital sign monitoring such as a pulse oximeter, to measure the oxygen saturation level. Another example is the TempTraq, a medical device realized in Ohio to monitor temperature through a sensor patch that can last up to 72 h and transmit real-time data wirelessly [67]. Other important instruments are app-based monitoring programs such as the Fitbit and Apple watch wearables, which extract heart rate and other health data, but also metabolic biosensors for continuous and non-invasive glucose analysis because of the severity of illness due to diabetes [68].

Dexamethasone is suggested for both severe and critically ill patients. If inflammatory markers are increased, tocilizumab, sarilumab, baricitinib, or tofacitinib should be considered in addition to steroids. Patients with contraindications to steroids could be treated with the association to baricitinib/remdesivir. Tocilizumab commonly causes a mild rise of transaminases [69], such as tofacitinib, sarilumab, and baricitinib [65]. 

Liver biochemical markers should be monitored in ambulatory patients to predict liver injury and in hospitalized patients with severe disease, to predict the risk of liver injury, drug-induced liver injury—DILI—and acute liver failure. Liver damage is usually cytolytic but a recent Indian study highlighted that AST and alkaline phosphatase are better indicators of COVID-19-induced liver injury. Thus, both cholestatic and cytolytic injury need to be assessed [70]. 

### 5.2. Telemedicine

EASL guidelines suggest lifestyle change, diet, and both aerobic and resistance training to reduce liver fat [4]. During the SARS-CoV-2 pandemic, telemedicine represented an important strategy to counsel patients’ behavior, their diet habits, and their physical activity. Lockdown changed the lifestyle of everyone, because of less availability of fresh and healthy food in some towns, increased intake of homemade “fatty food” such as sweets and pizza, and decreased intake of delivery food. Nevertheless, despite the closure of gyms, there was also a trend in some people carrying out more physical activity at home because of the increased free time [71,72,73]. 

Thus, some doctors take the chance to modify FLD patients’ behavior to take advantage of less lunch or dinner at restaurants, less availability of wine or cocktails, and more time to carry out physical activity. Telemedicine has represented in pandemic the main tool to monitor lifestyle change, which is the first line therapy for NAFLD.

A Spanish randomized trial on a cohort of 57 NAFLD subjects demonstrated that sending videos of physical exercise through WhatsApp or email, encouraging patients to walk around the garden through telephone as well as involving them in chat groups of motivation was very useful to obtain weight loss [74]. 

Another study evaluated telemedicine as a tool for dietary intervention in patients with NAFLD. People assigned to the intervention group, with video and/or telephone consultations, had less weight gain, highlighting the importance of telemedicine in the management of these patients [75].

Additionally, in Hershey, United States, three patients with biopsy-proven NASH had benefits thanks to a supervised 20-week telehealth-delivered exercise training program showing improvement through magnetic resonance imaging (MRI)-proton density fat fraction (PDFF) of measured liver fat, insulin resistance, body weight, and body fat in parallel with gains in physical fitness [76].

These studies show that telemedicine, through dietary programs with telephone counseling and fitness training, using videos and devices such as accelerometers on wrists to monitor the intensity of physical activity, could increase adherence and improve patients’ outcomes. Nowadays, it could be an important tool for people who cannot go to dietitians or personal trainers, and also for patients with limited economic possibilities.

### 5.3. COVID-19 Vaccination

Regarding vaccination in patients in FLD, the literature is very poor with incomplete information.

A Chinese multicenter study on 381 patients with pre-existing NAFLD, after two doses of inactivated vaccine against SARS-CoV-2 (Beijing Institute of Biological Products Co., Ltd., Beijing, China), highlighted a good immunogenicity with adequate titer of neutralizing antibodies in 95.5% of enrolled people. There was also a good safety profile because adverse reactions were mild and self-limiting. However, this study had some limitations such as the lack of a control group without hepatic steatosis to compare immunogenicity and the lack of information about NAFLD severity [77].

Another Japanese study on 295 patients with moderate/severe hepatic steatosis and a control group, compared the seroconversion rate after vaccination with two doses of BNT162b2 or CoronaVac. There was no difference in the seroconversion rate and adverse reactions. Throughout, there was a lower proportion of people with high titer response (cut-off: 160 for BNT162b2 and 20 for CoronaVac) among the FLD group than the control one, probably for immune dysfunction in people with CLD [78].

Of interest, a recent study comparing humoral responses to vaccination in patients with a different degree of CLD (MAFLD etiology patients were about 15% of population), demonstrated the absence of significant differences between cirrhotic and non-cirrhotic patients and the ability of the booster dose to induce a positive antibody titer in cirrhotic patients with a negative response to the primary vaccination course [3].

Further studies, with larger sample size, could better evaluate the efficacy of SARS-CoV-2 vaccination in FLD.

## 6. Conclusions

During the pandemic, SARS-CoV-2 infection and FLD were deeply interconnected: patients with FLD are more at risk of being infected while the pandemic and lockdown periods caused an increase in FLD incidence. In these patients, many risk factors are proven to be correlated with the severity of infection, particularly BMI, metabolic comorbidities, and liver fibrosis whereas there are still doubts if FLD per se is an independent risk factor.

There are also good sides to this situation: the need to limit outpatient visits and the subsequent implementation of telemedicine showed the possibility to achieve very satisfying results thanks to online and telephone counseling. Moreover, SARS-CoV-2 vaccination seems to be very effective in reducing the risk of severe infection and it has been demonstrated to have good safety and immunogenicity in these patients even if more studies are needed.

## Data Availability

Not applicable.
